# Contrasting roles for DNA methyltransferases and histone deacetylases in single-item and associative recognition memory

**DOI:** 10.1016/j.nepig.2017.02.001

**Published:** 2017-03-06

**Authors:** Hannah Scott, Anna E. Smith, Gareth R. Barker, James B. Uney, E. Clea Warburton

**Affiliations:** a School of Physiology, Pharmacology and Neuroscience, University of Bristol, Bristol BS8 1TD, UK; b School of Clinical Sciences, University of Bristol, Bristol BS8 1TD, UK

**Keywords:** Recognition memory, Perirhinal cortex, Prefrontal cortex, Hippocampus, DNMT, HDAC

## Abstract

Recognition memory enables us to judge whether we have encountered a stimulus before and to recall associated information, including where the stimulus was encountered. The perirhinal cortex (PRh) is required for judgment of stimulus familiarity, while hippocampus (HPC) and medial prefrontal cortex (mPFC) are additionally involved when spatial information associated with a stimulus needs to be remembered. While gene expression is known to be essential for the consolidation of long-term recognition memory, the underlying regulatory mechanisms are not fully understood. Here we investigated the roles of two epigenetic mechanisms, DNA methylation and histone deacetylation, in recognition memory. Infusion of DNA methyltransferase inhibitors into PRh impaired performance in novel object recognition and object-in-place tasks while infusions into HPC or mPFC impaired object-in-place performance only. In contrast, inhibition of histone deacetylases in PRh, but not mPFC, enhanced recognition memory. These results support the emerging role of epigenetic processes in learning and memory.

## 1 Introduction

Recognition memory is a fundamental process that enables us to judge whether we have encountered something before. It is considered to rely on multiple processes, depending on the type of information to be remembered including stimulus familiarity discrimination, the ability to judge the relative familiarity of a single item, stimulus location or associative recognition, which involves remembrance of a stimulus with its associated contextual information. The perirhinal cortex (PRh) is essential for familiarity discrimination (Ennaceur et al., 1996, Aggleton et al., 1997, Mumby et al., 2002a) while associative recognition involves interactions between the PRh and the hippocampus (HPC) and medial prefrontal cortex (mPFC) (Mitchell and Laiacona, 1998, Mumby et al., 2002b, Barker et al., 2007, Good et al., 2007, Barker and Warburton, 2011).

While long-term recognition memory formation has been shown to require expression of immediate early genes and a number of transcription factors (Zhu et al., 1995, Bozon et al., 2003, Warburton et al., 2005, Soulé et al., 2008, McNulty et al., 2012, Seoane et al., 2012, Barbosa et al., 2013, Beer et al., 2013), little is known about how these genes are regulated. Epigenetic regulation processes have been shown to be important for long term memory. For example, DNA methylation, the methylation of cytosines by DNA methyltransferases (DNMTs), is a transcriptional regulatory mechanism that has recently been implicated in single-item recognition memory (Morris et al., 2014, Tomizawa et al., 2015) and associative fear conditioning (Miller and Sweatt, 2007, Lubin et al., 2008, Halder et al., 2016). Furthermore DNMT inhibition in PRh or HPC has been shown to affect object-place associative memory (Mitchnick et al., 2015).

Another epigenetic mechanism strongly implicated in memory formation is the acetylation of lysine residues on histone tails in chromatin, a process mediated by histone acetyl transferases (HATs) and histone deacetylases (HDACs) (Graff and Tsai, 2013). Histone acetylation has been shown to be critical for the formation of fear and spatial memory (Guan et al., 2009, Sui et al., 2012) and transgenic mice in which HAT activity is inhibited showed significant impairments in spatial memory and novel object recognition. HAT inhibition in the PRh or the HPC was also found to impair object-place associative recognition memory (Mitchnick et al., 2016). Conversely, HDAC inhibition has been shown to rescue the deficit in object recognition memory observed after HAT mutation (Korzus et al., 2004) and to enhance object recognition memory following a shortened same phase which did not result in robust memory in control animals (Fontán-Lozano et al., 2008, Stefanko et al., 2009, Haettig et al., 2011).

In light of the clear evidence that both DNA methylation and histone acetylation are important for long-term memory, this study investigated the contribution of DNMTs and HDACs in the HPC, PRh and mPFC to long-term single-item and associative recognition memory, as there is little evidence currently available concerning the contribution of DNMTs and HDACs in discrete brain areas to recognition memory encoding and consolidation. Previous studies have shown that DNMT levels are increased during memory consolidation (Miller and Sweatt, 2007, Monsey et al., 2011, Morris et al., 2014). DNMT inhibitors, applied immediately after fear memory training could block conditioning-associated increases in gene methylation 1 h after the training session (Miller and Sweatt, 2007), suggesting that DNMTs exert functional effects during consolidation. In comparison, increased histone acetylation has been shown to commence during memory encoding in normal animals (Korzus et al., 2004, Fontán-Lozano et al., 2008, Wang et al., 2014). Hence, we hypothesised that inhibiting DNMT activity in vivo in the HPC, PRh or mPFC after the sample phase of a recognition memory task would impair recognition memory in the rat, and that conversely inhibiting HDAC activity in the PRh and mPFC before the sample phase would lead to enhancements in recognition memory.

## 2 Materials and methods

### 2.1 Animals

Adult male Lister Hooded rats were used for all experiments. Rats were three months old and weighing at least ~350 g at the time of surgery. All groups of animals undergoing the same cannulation procedure were littermates. Animals were not balanced for age among the various experiments, however all behavioural testing took place when rats were between three months and eleven months of age. Rats were housed in pairs under a 12 h light/dark cycle, with food and water available ad libitum. Experiments were performed during the dark phase. All animal procedures were performed in accordance with United Kingdom Animals Scientific Procedures Act (1986) and associated guidelines.

### 2.2 Surgery

Rats were implanted with bilateral cannulae aimed at the HPC, PRh or mPFC. To this end, rats were anaesthetised with isoflurane (4% for induction, 2–3% for maintenance) and placed into a stereotaxic frame to achieve a flat skull. Stainless-steel guide cannulae (26 gauge, Plastics One) were inserted through burr holes in the skull and fixed in place by stainless-steel skull screws (Plastics One) and bone cement. Cannula locations depended on whether cannulae were implanted individually or in combination with cannulae for a second brain region. HPC cannulae were inserted at: anterior-posterior (AP) − 4.2 mm, mediolateral (ML) ± 2.5 mm from bregma, dorsoventral (DV) − 3.0 mm from dura; or AP − 2.8 mm, ML ± 2.3 mm from bregma, DV − 3.5 mm from skull surface, cannula at an angle of 15° AP. PRh cannulae were implanted at AP − 5.6 mm, ML ± 4.5 mm from bregma, DV − 5.7 mm from skull surface, at an angle of 20° ML; or at AP − 5.6 mm, ML ± 4.5 mm from bregma, DV − 6.70 mm from skull surface, at an angle of 20° ML. For mPFC infusions, cannulae were inserted at AP + 3.20 mm, ML ± 0.75 mm, DV − 3.5 mm, all relative to bregma.

Following surgery, all rats received 5 ml saline s.c. for fluid replacements and 0.05 ml Vetergesic i.m. for analgesia and were allowed to recover in single housing for two weeks. Animals were then rehoused in pairs before behavioural procedures began. Obturators (Plastics One) were used to keep the cannulae patent in between infusions.

### 2.3 Infusions

20 mg/ml stock solutions of RG108 (Abcam, UK) and 5-aza-2′-deoxycytidine (5AZA; Sigma-Aldrich, UK) were made up in 0.08% acetic acid and in 100% DMSO, respectively. 15 mg/ml stock solutions of trichostatin A (TSA; Tocris, UK) were made up in 100% DMSO. Working solutions of 200 μg/ml (RG108 and 5AZA) or 0.6 mg/ml (for TSA) were made up fresh for each experiment by diluting with sterile 0.9% saline solution (Aqupharm No1, Animalcare Limited, UK). The drug concentrations chosen were based on effective doses used in previous studies (Miller and Sweatt, 2007, Zhao et al., 2010, Maddox and Schafe, 2011, Wang et al., 2014, Barbier et al., 2015) and on published IC50 values (Furumai et al., 2001, Khan et al., 2008). Rats were infused bilaterally into HPC, PRh or mPFC with either drug or the respective vehicle. For DNMT inhibitors RG108 and 5AZA, the infusion took place immediately after the sample phase of the task so as to examine effects on memory consolidation without disrupting memory encoding, as previous studies have identified a role for DNMTs during memory consolidation (Miller and Sweatt, 2007, Sui et al., 2012, Monsey et al., 2011). HDAC inhibitor TSA was infused 15 min prior to the start of the sample phase to ensure the drug exerts its effects during memory encoding, in line with previous studies that found changes in histone acetylation during memory encoding (Korzus et al., 2004, Fontán-Lozano et al., 2008, Wang et al., 2014). For the infusions a 33 gauge cannula (Plastics One) was attached to a 0.025 ml Hamilton syringe via polyethylene tubing and inserted into the implanted cannula. Infusions were performed over 2 min at 0.25 μl/min into HPC and at 0.5 μl/min into cortical areas. The volumes used have been shown to achieve a drug spread of approximately 0.5–2.5 mm from the injection site and were shown to be restricted to the PRh for intraperirhinal infusions (Martin, 1991, Attwell et al., 2001, Barker and Warburton, 2015). Infusion cannulae where left in situ for 5 min after the end of infusion to allow for diffusion of the infusate. All experiments were run using a cross-over design and each animal was re-tested following a minimum 3 days rest period.

### 2.4 Apparatus

All behavioural testing took place in a wooden, open-topped arena (50 × 90 × 100 cm), the floor of which was covered with sawdust. For the novel object recognition (NOR) task, all four walls were grey and the arena was surrounded with a black cloth up to a height of 1.5 m to conceal the experimenter. For the object-in-place (OIP) task, one of the walls was substituted with a black wall and the curtains were removed from two sides of the arena to provide extra-maze cues. The rats' behaviour was recorded via an overhead camera and a computer. Objects used in the behavioural tasks were made out of Duplo® (Lego UK, Slough, UK) in varying size, shape and colour. The objects were cleaned with 100% ethanol before each run to eliminate olfactory cues.

### 2.5 Behavioural testing

**Fig. 1:**
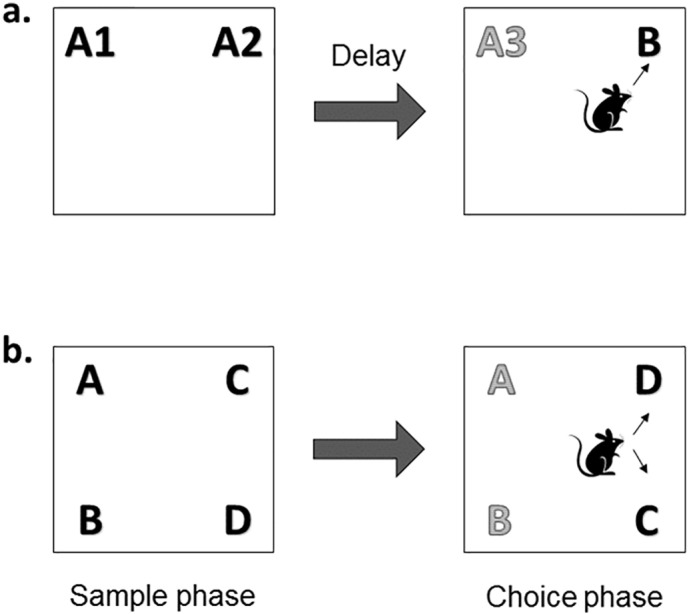
Schematic representation of object recognition tasks. In the NOR task (a) normal rats are expected to spend more time exploring the novel object (B) during the choice phase. In the OIP task (b) normal rats spend more time exploring the object pair in the novel configuration (C and D) compared to the pair that has remained in the same locations.

Before object recognition testing began, rats were habituated to the arena for 5 min daily for 5 days and handled regularly. Rats were subjected to two tasks – the novel object recognition (NOR) and the object-in-place (OIP) tasks (see Fig. 1). Each task consisted of a sample phase of variable length and a choice phase of 180 s length separated by a delay of 24 h, which the rat spent in its home cage.

#### 2.5.1 NOR task

For the sample phase two identical copies of an object (objects A1 and A2) were placed in adjacent corners of the arena (see Fig. 1a). The rat was placed in the arena facing the centre of the opposite wall and was allowed to explore the objects for 40 s or until it had spent a total of 240 s in the arena, whichever occurred soonest (‘standard sample phase’). For the histone deacetylation experiments, in which we hypothesised that an enhancement of memory performance might be observed, we also used a sample phase in which the rat was allowed a maximum cumulative object exploration of 20 s or a total of 120 s in the arena (‘subthreshold sample phase’). Following a 24 h delay, memory performance was assessed in a choice phase of 180 s. During this phase a third copy of object A (A3) and a novel object (object B) were placed into the arena (Fig. 1a). The position of the novel object (left or right) and the objects used as novel or familiar were counterbalanced across animals.

#### 2.5.2 OIP task

In the sample phase, four different objects (A, B, C and D) were placed 10 cm from the corners of the arena (Fig. 1b). Each rat was allowed to explore the objects for 300 s. In the histone deacetylation experiments, a subthreshold sample phase was used, wherein animals were allowed a maximum cumulative object exploration of 25 s or a total of 120 s in the arena. After the 24 h delay, recognition memory performance was assessed in a test phase of 180 s duration in which the animal was placed back into the arena, which contained objects A, B, C and D, but two of the objects (e.g. C and D) had exchanged positions (the moved objects) while the other objects (A and B) remained in the same position (the unmoved objects) (Fig. 1b). The position (left or right) of the object pairs during the sample phase and the moved object pair in the test phase were counterbalanced between rats.

### 2.6 Statistical analysis

The time spent exploring the novel and familiar objects in the NOR choice phase or the unmoved and moved objects in the OIP choice phase was recorded with the experimenter blind to the drug status of each rat. Exploration was defined as the animal directing its nose towards the object within a distance of 2 cm. The discrimination ratio was calculated as the difference in time between exploring the novel and familiar objects (or object-place pairings) divided by total exploration time. Groups were compared using two-way, repeated-measures analysis of variance (ANOVA). The within-subjects factor was infusion (vehicle vs drug) for both experiments and the between-subjects factor was drug type (RG108 vs 5AZA) for the DNA methylation experiments and sample phase type (subthreshold or standard) for the histone acetylation experiments. Bonferroni-corrected multiple comparisons were performed where applicable and one-sample *t*-tests against a discrimination ratio of zero (equal exploration of novel and familiar object) were used to analyse whether the animals had discriminated between the objects. For all statistical analyses a significance level of 0.05 was used.

### 2.7 Histology

**Fig. 2:**
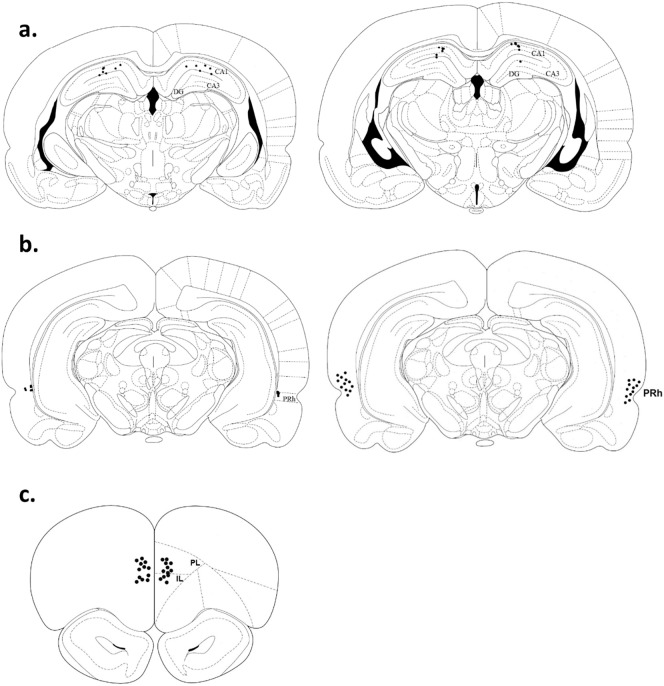
Placement of infusion cannulae. a. Infusion cannulae tips (black dots) were located in the dorsal hippocampus (bregma − 4.2 mm, − 3.8 mm; a), in the perirhinal cortex (bregma − 5.6 mm, − 4.80 mm; b) and in the mPFC (bregma + 4.20 mm; c). Several of the dots overlap. Images of coronal sections adapted from Paxinos and Watson (1998).

At the end of the behavioural experiments, the rats were anaesthetised with Euthatal (Rhȏne Mérieux) and perfused transcardially with 0.1 M phosphate buffer (pH 7.4), followed by 4% paraformaldehyde. After postfixation in paraformaldehyde for 24 h the brain was incubated in 30% sucrose in 0.2 M phosphate buffer for 48 h. Coronal sections were cut at 40 μm on a cryostat, mounted onto slides and stained with cresyl violet. Correct placement of the cannulae was confirmed under a light microscope using a rat brain atlas (Paxinos and Watson, 1998). Cannula tips of all rats were confirmed to be present in dorsal HPC, PRh and mPFC, as expected (Fig. 2).

## 3 Results

### 3.1 Infusion of RG108 or 5AZA into the HPC impaired object-in-place but not novel object recognition memory

**Fig. 3:**
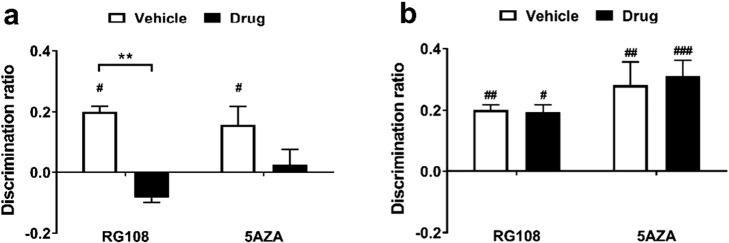
Effect of DNMT inhibition in the HPC on consolidation of OIP and NOR memory. Rats were infused into the HPC with RG108 or 5AZA immediately after the sample phase of an OIP (a; RG108: *n* = 13; 5AZA: *n* = 20) or a NOR task (b; RG108: *n* = 12; 5AZA: *n* = 13) with a 24 h delay. The mean discrimination ratio (± s.e.m.) is shown. *n* numbers given apply to both drug and respective control group. Age range of rats at time of experiment: 3–11 months. Statistical differences between infusion groups, ^⁎⁎^*p* < 0.01. Significance from discrimination ratio of zero, ^#^*p* < 0.05, ^##^*p* < 0.01, ^###^*p* < 0.001.

DNMT inhibitors RG108 or 5AZA were infused into the HPC immediately after the sample phase of an OIP task. Compared to vehicle-infused rats, rats that had been administered RG108 or 5AZA showed reduced discrimination in the OIP task (Fig. 3a). Repeated-measures ANOVA showed a significant main effect of infusion (*F*_1,30_ = 15.6, *p* = 0.0004). No significant effect of drug type (*F*_1,30_ = 0.257, *p* = 0.616) and no significant infusion × drug type interaction (*F*_1,30_ = 2.11, *p* = 0.157) was observed. *Post-hoc* comparisons revealed that performance after RG108 infusion was significantly different to performance after vehicle infusion (*t*_11_ = 3.42, *p* = 0.004) while performance of 5AZA-infused rats was not significantly different compared to control rats (*t*_19_ = 2.039, *p* = 0.1006). Furthermore, rats infused with vehicle showed significant discrimination between the novel and familiar configurations (RG108, *t*_11_ = 3.04, *p* = 0.011; 5AZA, *t*_19_ = 2.66, *p* = 0.016) whereas performance in the task was disrupted after infusion of either RG108 (*t*_11_ = − 1.58, *p* = 0.143) or 5AZA (*t*_19_ = 0.54, *p* = 0.59).

Next the effect of DNMT inhibition was tested in the NOR task. As can be seen in Fig. 3b, rats treated with vehicle or either of the DNMT inhibitors were able to discriminate in the task. Two-way ANOVA showed no significant main effect of infusion (*F*_1,23_ = 0.018, *p* = 0.893) or drug type (*F*_1,23_ = 4.14, *p* = 0.054) and no significant infusion × drug type interaction (*F*_1,23_ = 0.042, *p* = 0.839). Vehicle-infused rats showed significant discrimination between novel and familiar objects (RG108, *t*_11_ = 3.41, *p* = 0.006; 5AZA, *t*_11_ = 4.36, *p* = 0.001), as did rats that had received RG108 (*t*_11_ = 2.34, *p* = 0.039) or 5AZA (*t*_11_ = 5.80, *p* = 0.0001).

The effect of RG108 or 5AZA infusion on total exploratory behaviour during the choice phase was also analysed (Table 1). No differences in the total amount of exploration during the OIP task were observed, confirmed by a non-significant main effect of infusion (*F*_1,30_ = 0.578, *p* = 0.453), a non-significant effect of drug type (*F*_1.30_ = 0.034, *p* = 0.854) and a non-significant infusion × drug type interaction (*F*_1,30_ = 0.027, *p* = 0.872). In the NOR task, there was no significant main effect of infusion (*F*_1,23_ = 0.992, *p* = 0.812) and no significant infusion × drug type interaction (*F*_1,23_ = 0.547, *p* = 0.467) however there was a difference in the total amount of object exploration between the two types of drug (main effect of drug type, *F*_1,23_ = 122.7, *p* < 0.0001). Further analyses revealed that the behaviour of vehicle-infused rats in the 5AZA experiment was significantly different to behaviour of rats infused with RG108 (*t*_46_ = 7.64, *p* < 0.0001) or corresponding vehicle (*t*_46_ = 6.50, *p* < 0.0001).

These results indicate that infusion of DNMT inhibitor RG108 or 5AZA into the HPC immediately after the sample phase significantly impaired OIP but not NOR performance.

### 3.2 Infusion of RG108, but not 5AZA, into the PRh impaired novel object recognition and object-in-place memory

**Fig. 4:**
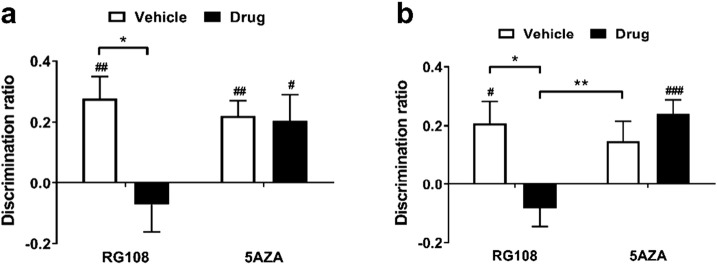
Effect of DNMT inhibition in the PRh on consolidation of NOR and OIP memory. Immediately after the sample phase of a NOR (a; RG108: *n* = 9; 5AZA: *n* = 12) or an OIP task (b; RG108: *n* = 8; 5AZA: *n* = 12), rats received a RG108 or 5AZA infusion into the PRh. The mean discrimination ratio (± s.e.m.) after a 24 h delay is shown. *n* numbers given apply to both drug and respective control group. Age range of rats at time of experiment: 3–11 months. Statistical differences between infusion groups, ^⁎^*p* < 0.05, ^⁎⁎^*p* < 0.01. Significance from discrimination ratio of zero, ^#^*p* < 0.05, ^##^*p* < 0.01, ^###^*p* < 0.001.

The effect of intra-perirhinal infusion of RG108 or 5AZA on NOR memory performance can be seen in Fig. 4a. Statistical analysis revealed a significant main effect of infusion (*F*_1,19_ = 6.03, *p* = 0.024) and a significant infusion × drug type interaction (*F*_1,19_ = 5.06, *p* = 0.037). Further analysis confirmed that RG108-infused animals were significantly impaired in the NOR task compared to rats that had received vehicle (*t*_8_ = 3.11, *p* = 0.012). While vehicle-infused rats were able to discriminate between the novel and the familiar object (RG108 experiment, *t*_8_ = 3.86, *p* = 0.005; 5AZA experiment, *t*_11_ = 4.40, *p* = 0.001), only rats infused with the inhibitor 5AZA showed significant discrimination in the task (R108, *t*_8_ = 0.81, *p* = 0.44; 5AZA, *t*_11_ = 5.08, *p* = 0.034).

Performance in the OIP task was impaired after post-sample infusion of RG108, but not 5AZA, into the PRh (Fig. 4b). A significant main effect of drug type (*F*_1,18_ = 6.07, *p* = 0.024) and a significant infusion × drug type interaction (*F*_1,18_ = 6.80, *p* = 0.018) was observed. Furthermore there was a significant difference in discrimination between RG108 and vehicle-treated rats (*t*_7_ = 2.55, *p* = 0.040). Rats that had received vehicle showed significant discrimination between the novel and the familiar object in the RG108 experiment (*t*_7_ = 2.83, *p* = 0.025) while in the 5AZA experiment vehicle-infused rats were able to discriminate between the two objects but this did not reach statistical significance (*t*_11_ = 2.14, *p* = 0.06). Rats infused with 5AZA, but not RG108, were able to discriminate in the OIP task (RG108, *t*_7_ = 1.36, *p* = 0.22; 5AZA, *t*_11_ = 5.07, *p* = 0.0004).

No differences in total amount of exploration between vehicle and DNMT inhibitor infusions were observed in either task (Table 1), confirmed by a non-significant main effect of infusion (NOR, *F*_1,19_ = 1.01, *p* = 0.327; OIP, *F*_1,18_ = 2.64, *p* = 0.954) and a non-significant infusion × drug type interaction (NOR, *F*_1,19_ = 0.840, *p* = 0.371; OIP, *F*_1,18_ = 1.38, *p* = 0.256). A significant main effect of drug type was observed in the OIP task only (NOR, *F*_1,19_ = 3.39, *p* = 0.081; OIP, *F*_1,18_ = 207.1, *p* < 0.0001). Specifically, there was a significant difference in exploration behaviour between vehicle-infused rats from the 5AZA experiment and drug (*t*_6_ = 8.94, *p* < 0.0001) or vehicle-treated rats in the RG108 experiment (*t*_6_ = 6.99, *p* < 0.0001).

Thus, infusion of RG108 into the PRh significantly impaired performance in both the NOR and OIP tasks while infusion of 5AZA in the PRh did not affect performance in the NOR or the OIP task.

### 3.3 Infusion of RG108 or 5AZA into the mPFC reduced performance in the object-in-place task

**Fig. 5:**
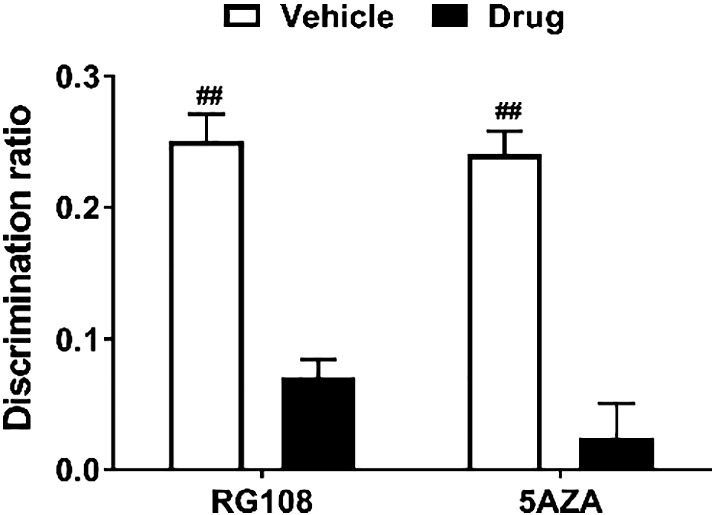
Effect of DNMT inhibition in the mPFC on an OIP memory task. Rats were infused with RG108 (*n* = 12) or 5AZA (*n* = 12) into the mPFC immediately after the sample phase of an OIP task. The mean discrimination ratio achieved during the choice phase after a 24 h delay is depicted (+ s.e.m.). *n* numbers given apply to both drug and respective control group. Age range of rats at time of experiment: 3–11 months. Significance from discrimination ratio of zero, ^##^*p* < 0.01.

Rats were infused with either RG108 or 5AZA into the mPFC and tested on the OIP task only as it has been demonstrated extensively that the mPFC is not required for NOR memory (Ennaceur et al., 1997, Mitchell and Laiacona, 1998, Hannesson et al., 2004, Barker et al., 2007). As shown in Fig. 5, discrimination in the OIP task was reduced after RG108 or 5AZA infusion compared to vehicle administration. Statistical analysis indicated a significant main effect of infusion (*F*_1,22_ = 5.651, *p* = 0.027) but no significant main effect of drug type (*F*_1,22_ = 0.151, *p* = 0.701) and no significant infusion × drug type interaction (*F*_1,22_ = 0.035, *p* = 0.852). Further analysis revealed that neither RG108 nor 5AZA-infused animals were significantly impaired in the OIP task compared to rats that had received vehicle (RG108, *t*_11_ = 1.85, *p* = 0.202; 5AZA, *t*_11_ = 4.61, *p* = 0.055). While vehicle-infused rats were able to discriminate between the moved and the unmoved objects (RG108 experiment, *t*_11_ = 3.757, *p* = 0.003; 5AZA experiment, *t*_11_ = 4.09, *p* = 0.002), rats infused with RG108 or 5AZA did not show significant discrimination (RG108, *t*_11_ = 0.731, *p* = 0.480; 5AZA, *t*_11_ = 0.27, *p* = 0.793).

The total amount of exploration in the choice phase did not differ between vehicle and RG108 or 5AZA infusions into the mPFC (Table 1), as there were no significant main effects of either infusion (*F*_1,22_ = 0.168, *p* = 0.685) or drug type (*F*_1,22_ = 0.481, *p* = 0.495) and a non-significant infusion × drug type interaction was observed (*F*_1,22_ = 2.166, *p* = 0.155).

Taken together, the results suggest that both RG108 and 5AZA infusions into the mPFC cause a reduction in the ability of the rat to discriminate in the OIP task.

### 3.4 Infusion of TSA into the PRh led to robust object recognition memory after a subthreshold sample phase

**Fig. 6:**
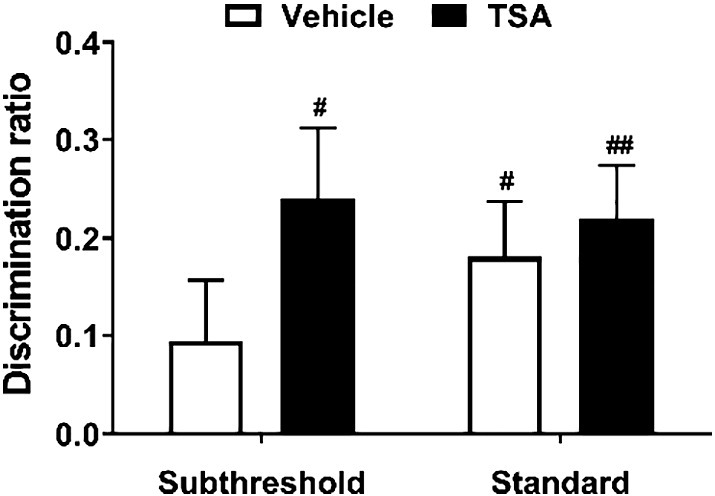
Effect of HDAC inhibition in the PRh on encoding of NOR memory. Immediately before the standard (*n* = 12) or subthreshold (*n* = 12) sample phase of a NOR task, rats received an infusion of either TSA or vehicle into the PRh. The mean discrimination ratio (+ s.e.m.) after a 24 h delay is shown. *n* numbers given apply to both drug and respective control group. Age range of rats at time of experiment: 3–11 months. Significance from discrimination ratio of zero, ^#^*p* < 0.05, ^##^*p* < 0.01.

To inhibit histone deacetylation, TSA was infused into the PRh 15 min before the start of the sample phase of a NOR task. As can be seen in Fig. 6, animals that received TSA infusion showed normal performance in both subthreshold and standard sample phase versions of the NOR task, whereas vehicle-infused animals did not show significant discrimination when the subthreshold sample phase was used. Two-way ANOVA showed no significant main effect of infusion (*F*_1,22_ = 1.43, *p* = 0.244) or sample phase type (*F*_1,22_ = 0.306, *p* = 0.586), and no significant infusion × sample phase type interaction (*F*_1,22_ = 0.482, *p* = 0.495). Vehicle-infused rats showed significant discrimination between novel and familiar objects after a standard (*t*_11_ = 2.88, *p* = 0.015) but not subthreshold (*t*_11_ = 1.33, *p* = 0.210) sample phase. Conversely, TSA-infused animals showed significant discrimination after both sample phase types (subthreshold *t*_11_ = 2.98, *p* = 0.013; standard *t*_11_ = 3.64, *p* = 0.004).

The effect of TSA infusion on total exploratory behaviour in the choice phase was also analysed (Table 2). There was no significant main effect of either infusion (*F*_1,22_ = 1.65, *p* = 0.212) or sample phase type (*F*_1,22_ = 10.3, *p* = 0.263), and no significant infusion × sample phase type interaction (*F*_1,22_ = 0.873, *p* = 0.360).

These results suggest that TSA infusions into the PRh lead to robust long-term object recognition memory, even after a subthreshold sample phase.

### 3.5 Infusion of TSA into the mPFC had no effect on object-in-place associative recognition memory

**Fig. 7:**
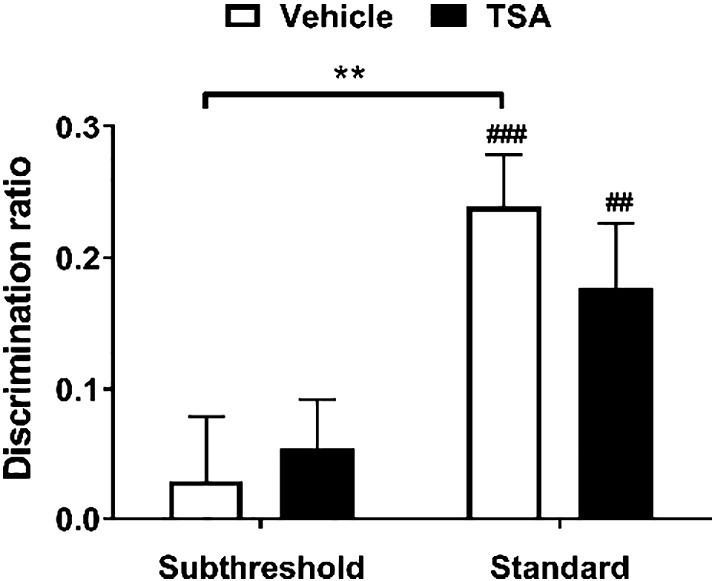
Effect of HDAC inhibition in the mPFC on an OIP memory task. Rats were infused with TSA into the mPFC immediately before the standard (*n* = 12) or subthreshold (*n* = 12) sample phase of an OIP task. The mean discrimination ratio (+ s.e.m.) after a 24 h delay is shown. *n* numbers given apply to both drug and respective control group. ​Age range of rats at time of experiment: 3–11 months. Statistically significant differences between sample phase types, **p < 0.01. Significance from discrimination ratio of zero, ^##^*p* < 0.01, ^###^*p* < 0.001.

To investigate the role of histone acetylation in associative recognition memory, TSA was infused into the mPFC 15 min prior to the sample phase of the OIP task. As is shown in Fig. 7, TSA infusion did not influence performance in either the standard or the subthreshold versions of the OIP task. Statistical analysis found no significant main effect of infusion (*F*_1,22_ = 0.126, *p* = 0.726), but did reveal a main effect of sample phase type (*F*_1,22_ = 12.4, *p* = 0.002). However, there was no significant infusion × sample phase type interaction (*F*_1,22_ = 0.694, *p* = 0.414). Further analysis using paired *t*-tests indicated that in the OIP task vehicle-infused animals performed significantly better after a standard sample phase compared to a subthreshold sample phase (*t*_22_ = − 2.95, *p* = 0.007), whereas for TSA-infused animals this difference was not significant (*t*_22_ = − 1.76, *p* = 0.093). After a standard sample phase, both vehicle- (*t*_11_ = 5.35, *p* < 0.0005) and TSA-infused (*t*_11_ = 3.19, *p* = 0.009) animals showed significant discrimination between familiar and novel object-location combinations. Conversely, after a subthreshold sample phase neither vehicle (*t*_11_ = 0.516, *p* = 0.616) nor TSA-infused (*t*_1,11_ = 1.27, *p* = 0.230) animals showed significant discrimination.

Analysis of total object exploration in the choice phase (Table 2) revealed no significant main effect of either infusion (*F*_1,22_ = 0.031, *p* = 0.861) or sample phase type (*F*_1,22_ = 0.083, *p* = 0.776), and no statistically significant infusion × sample phase type interaction (*F*_1,22_ = 0.845, *p* = 0.368).

Together these results suggest that infusion of TSA into the mPFC does not affect performance in the OIP task after either a subthreshold or standard sample phase.

## 4 Discussion

In the present study we investigated the roles of two epigenetic mechanisms, DNA methylation and histone acetylation, in recognition memory function. By using targeted infusions of inhibitors against DNMTs or HDACs, we have identified that DNA methylation and histone deacetylation play contrasting roles in recognition memory.

### 4.1 The role of DNMTs in recognition memory

We have shown for the first time that DNA methylation in the PRh is required for single-item recognition memory and that DNA methylation in the mPFC is required for object-place associative recognition memory. In agreement with previous studies (Mitchnick et al., 2015), we have also found that associative recognition memory relies on DNMT function in the PRh and in the HPC.

Taken together the results suggest that DNA methylation is essential for the familiarity discrimination component of recognition memory (which relies on the integrity of the PRh) as well as for the integration with the associative component of recognition memory (which is dependent on both the HPC and mPFC). The consolidation of single-item and associative recognition memory are both supported by changes in synaptic strength as long-term depression (LTD) is the predominant mechanism underlying familiarity discrimination in the PRh (Brown and Xiang, 1998, Griffiths et al., 2008), while it is long-term potentiation (LTP) in the HPC and mPFC that has been found to play a role in recognition memory consolidation (Clarke et al., 2010, Xiang and Brown, 2004, Hirsch and Crepel, 1991). In order for these synaptic plasticity processes to facilitate the formation of long-term memories, long-lasting biochemical alterations at the synapse level, mediated by gene expression changes are also required. LTP has previously been shown to be associated with DNA methylation in HPC and mPFC (Feng et al., 2010, Levenson et al., 2006, Miller and Sweatt, 2007, Morris et al., 2014, Sui et al., 2012). The observation that DNMT function in the PRh is required for familiarity discrimination, suggests that DNA methylation may also be linked to LTD in the PRh. Hence DNA methylation may have a more general role in recognition memory function, independent of brain region or underlying plasticity process. Since DNA methylation at gene promoters has been associated with transcriptional repression (Brenet et al., 2011), DNMT inhibition may impair recognition memory consolidation by inhibiting methylation of genes known to be negative regulators of memory formation, such as protein phosphatase 1 and calcineurin (Miller and Sweatt, 2007, Miller et al., 2010). Alternatively, the DNMT inhibitor may exert its effect by inhibiting the exonic methylation of plasticity genes, thereby affecting production of alternative transcripts (Lubin et al., 2008) and reducing the capability of the neuron to ‘fine-tune’ its neuronal properties.

Two different types of DNMT inhibitor, RG108 and 5AZA, were used in this study. Both drugs produced comparable effects when infused into the HPC or mPFC. In contrast, only RG108, and not 5AZA, infusion into the PRh caused impairment in the recognition memory tasks. It should be mentioned that vehicle-infused rats in the 5AZA experiment did not show significant discrimination in the OIP task. However, the significant discrimination of rats infused with 5AZA along with the lack of a significant difference between performances in the task by vehicle or 5AZA-infused rats, supports the interpretation that 5AZA infusion, in contrast to RG108 infusion, did not impair performance in either recognition memory task. DNMT inhibitor 5AZA is a cytosine analogue that blocks DNMT function by covalently binding to the enzyme's catalytic cysteine residue (Santi et al., 1983, Santi et al., 1984) and may require incorporation into the DNA strand to exert its inhibitory effect (Creusot et al., 1982, Stresemann et al., 2006). In contrast, RG108 is a non-nucleoside inhibitor that acts by reversibly blocking DNMT's active site, without requiring incorporation into the DNA strand (Brueckner et al., 2005). In addition, RG108 is considered to be less likely to show non-specific effects, as it was modelled specifically to fit the active pocket of DNMT1 (Brueckner et al., 2005). While it is clear that the two inhibitors may have different mechanisms of action it is of great interest to establish the mechanistic and functional differences between them in order to elucidate the differential effects on DNMT function in the PRh.

### 4.2 The role of HDACs in recognition memory

This is the first study demonstrating that direct infusion of a general HDAC inhibitor into the PRh can lead to robust object recognition memory after a subthreshold sample phase in the NOR task. This result concurs with previously published observations in which systemic injections of TSA enhanced object recognition memory after a subthreshold sample phase (Fontán-Lozano et al., 2008). No enhancement in memory was seen following a standard sample phase with TSA infusion. This result is in agreement with findings from other researchers in which HDAC inhibition with either TSA or sodium butyrate had no effect on object recognition memory after a standard sample phase (Korzus et al., 2004, Stefanko et al., 2009).

In contrast to the improvement in memory function seen when HDACs were inhibited in the PRh in the object recognition task, infusion of TSA into the mPFC did not lead to robust memory of object-place associations in the OIP task after a subthreshold sample phase. However, other studies have shown that increases in histone acetylation in the mPFC enhanced fear extinction (Bredy et al., 2007) and trace fear memory (Sui et al., 2012). It is possible therefore that the effects of histone acetylation in memory may be task-specific, however a recent study using the OIP task found that HAT inhibition in the HPC and PRh impaired associative recognition memory (Mitchnick et al., 2016), indicating that histone acetylation in other brain areas is required for OIP task performance.

Taken together, these findings suggest that histone acetylation enhances the PRh-dependent familiarity discrimination component of recognition memory, but does not affect the mPFC-dependent integration of object and place associations in recognition memory.

HDACs have previously been shown to be involved in mediating the changes in synaptic strength thought to underlie memory. Application of HDAC inhibitors enhanced LTP in hippocampal, prefrontal and amygdala slices (Alarcon et al., 2004, Levenson et al., 2004, Yeh et al., 2004, Vecsey et al., 2007, Miller et al., 2008, Sui et al., 2012). Our finding that HDAC inhibitor infusion into the PRh enhances object recognition memory suggests that HDACs mediate synaptic plasticity in this brain region. However, there are as of yet no published studies investigating this mechanism. In other brain areas, studies of LTD, the mechanism thought to underlie familiarity discrimination in the PRh (Brown and Xiang, 1998, Griffiths et al., 2008), have yielded conflicting results, finding alternately that HDAC inhibitor may have no effect on (Alarcon et al., 2004) or may even block (Guan et al., 2002, Hanson et al., 2013) LTD. Further study is therefore required to determine whether HDAC inhibition in the PRh produces an enhancement in LTD concurrent with the enhancement observed in object recognition memory. We have shown that HDAC inhibition, which has been shown to enhance LTP in acute prefrontal slices (Sui et al., 2012), did not enhance associative recognition memory. These studies point towards a selective role of histone deacetylation in recognition memory function, which is task dependent and may or may not be directly coupled to underlying mechanisms of synaptic plasticity.

The enhancement observed in familiarity discrimination following TSA infusion into the PRh is in line with the molecular brake pad hypothesis, which states that HDACs act as ‘brakes’, opposing the action of constitutively active HATs (McQuown and Wood, 2011). In McQuown and Wood's model, HDAC inhibitors remove the HDAC ‘brakes’, leading to an increase in HAT-mediated histone acetylation at the promoters of plasticity-related genes, thereby increasing the expression of these genes, leading to enhanced synaptic plasticity and memory. In animal studies, inhibition of HDAC activity has been shown to increase the acetylation and expression of genes including *Arc*, *c-Fos*, *Bdnf*, *Egr-1*, *Camk2a*, *Creb1* and *Gria1*, concomitant with enhancements in memory (Guan et al., 2009, Graff et al., 2014). Future studies using inhibitors that are selective for specific classes of HDACs will provide a more detailed understanding of the contribution of different HDAC subtypes to recognition memory.

Recently HDACs have been reported to deacetylate non-histone substrates including transcription factors, signalling proteins and RNA binding proteins (Spange et al., 2009). In addition to their epigenetic function HDACs could therefore also play a part in recognition memory formation and consolidation by regulating additional factors involved in transcription and RNA processing.

## 5 Conclusion

Increases in DNMT expression and histone acetylation have been seen to occur concomitantly as a consequence of LTP or memory formation in various brain regions and tasks (Monsey et al., 2011, Sui et al., 2012). In fact, DNMTs may interact directly with HDACs (Fuks et al., 2001, Ling et al., 2004) and DNA methylation sites can act as a recruitment point for HDACs, which induce modification of chromatin structure and further transcriptional repression (Jones et al., 1998, Ng et al., 1999). Infusions of the HDAC inhibitor TSA are able to ameliorate impairments in memory and synaptic plasticity seen as a result of DNMT inhibition in HPC- and amygdala-dependent tasks (Miller et al., 2008, Maddox and Schafe, 2011, Monsey et al., 2011). The presented results support the hypothesis that both DNA methylation and histone acetylation play crucial roles in the regulation of gene changes that underlie recognition memory in the PRh. Further experiments in which DNMT and HDAC inhibitor infusions are performed together would need to be carried out to investigate whether DNA methylation and histone acetylation work in concert to produce perirhinal recognition memory.

## Conflicts of interest

The authors declare no conflicts of interest.

## Figures and Tables

**Table 1:** Total exploration in the NOR and the OIP tasks after DNMT inhibition. Mean exploration time ± s.e.m. during the choice phase is shown for rats infused with either vehicle or drug (RG108 or 5AZA) into the HPC, PRh or mPFC.

	Task	Drug	Total exploration (s)	
			Vehicle	Drug
HPC	NOR	RG108	63.32 (4.08)	69.41 (4.43)
		5AZA	33.68 (1.83)	34.58 (2.06)
	OIP	RG108	74.24 (4.15)	77.69 (4.48)
		5AZA	73.82 (4.12)	76.05 (4.80)
PRh	NOR	RG108	50.31 (6.03)	40.05 (5.23)
		5AZA	39.37 (2.89)	38.89 (3.00)
	OIP	RG108	90.25 (5.82)	103.84 (4.21)
		5AZA	49.49 (2.78)	51.68 (3.19)
mPFC	OIP	RG108	77.85 (5.06)	86.24 (4.28)
		5AZA	88.03 (4.82)	83.30 (5.20)

**Table 2:** Total exploration in the NOR and OIP tasks after HDAC inhibition. Mean exploration time ± s.e.m. during the choice phase is shown for rats infused with either vehicle or TSA. Infusions were made into the PRh and rats were subjected to the NOR task while rats infused in the mPFC underwent OIP testing.

	Task	Sample phase	Total exploration (s)	
			Vehicle	TSA
PRh	NOR	Subthreshold	24.47 (1.95)	30.52 (4.17)
		Standard	25.19 (2.67)	26.14 (3.75)
mPFC	OIP	Subthreshold	37.27 (1.94)	34.58 (1.74)
		Standard	34.38 (2.09)	36.20 (3.28)
